# Patterns of Sexually Transmitted Co-infections and Associated Factors Among Men Who Have Sex With Men: A Cross-Sectional Study in Shenyang, China

**DOI:** 10.3389/fpubh.2022.842644

**Published:** 2022-05-31

**Authors:** Ze-Hao Ye, Shuo Chen, Fan Liu, Si-Tong Cui, Zhao-Zhen Liu, Yong-Jun Jiang, Qing-Hai Hu

**Affiliations:** ^1^National Health Commission Key Laboratory of AIDS Immunology, National Clinical Research Center for Laboratory Medicine, The First Affiliated Hospital of China Medical University, Shenyang, China; ^2^Key Laboratory of AIDS Immunology, Chinese Academy of Medical Sciences, Shenyang, China; ^3^Key Laboratory of AIDS Immunology of Liaoning Province, Shenyang, China; ^4^Collaborative Innovation Center for Diagnosis and Treatment of Infectious Diseases, Hangzhou, China

**Keywords:** MSM, STIs, co-infection, pattern, China

## Abstract

**Background:**

Men who have sex with men (MSM) are disproportionately affected by sexually transmitted infections (STIs). We sought to describe patterns of sexually transmitted co-infections and explore factors associated with increased acquisition of STIs among MSM.

**Methods:**

We enrolled MSM in Shenyang, China, between July and December 2020 to test for four STIs, including human papillomavirus (HPV), *Chlamydia trachomatis* (CT), *Neisseria gonorrhoeae* (NG), and *Treponema pallidum* (TP). Data regarding demographic and behavioral characteristics of participants were collected through a self-administered digital questionnaire. We adopted the ordinal logistic regression model to identify factors associated with acquiring more STIs.

**Results:**

Overall, 177 participants with completed test results for all four STIs were analyzed. These participants had a median age of 29.0 (interquartile range: 23.0–38.0) years. The prevalence of STI co-infections was 23.7% [42/177; 95% confidence interval (CI), 17.8%−30.8%], among which HPV/CT (47.1%) and HPV/CT/NG (50.0%) co-infection were the predominant types among participants with dual and multiple infections, respectively. Participants who had a higher educational background [adjusted odds ratio (aOR), 0.46; 95% CI, 0.24–0.85; *P* = 0.014] and had a history of STIs (aOR, 2.53; 95% CI, 1.24–5.18; *P* = 0.011) were positively associated with acquiring more STIs.

**Conclusions:**

MSM in Shenyang suffer a substantial burden of sexually transmitted co-infections. An optimized multi-STI integration strategy targeting prevention, surveillance, screening, and treatment is warranted to reduce the prevalence of sexually transmitted co-infections, especially in less-educated MSM.

## Introduction

Sexually transmitted infections (STIs) are acquired by more than one million individuals daily worldwide (1). Though not usually fatal, STIs exert a substantial disease burden (2). Certain viral STIs, such as human papillomavirus (HPV) can resist eradication and thus persist for life. Despite being curable with antibiotics, some bacterial STIs are usually asymptomatic and consequently readily progress to symptoms affecting the urethra or spread within the body [e.g., *Chlamydia trachomatis* (CT) and *Neisseria gonorrhoeae* (NG)] (3), whereas others, such as *Treponema pallidum* (TP), can cause severe neurological complications if ignored (4). In addition to related symptoms, these STIs may promote re-acquisition of other STIs (5, 6) or human immunodeficiency virus (HIV) (7, 8) through inflammation or ulceration. STI co-infections are reportedly associated with a higher proportion of inflammation (9), which in turn can increase an individual's STI burden and increase the chances of HIV acquisition.

Men who have sex with men (MSM) are disproportionately affected by STIs. Because of their sexual network or behavioral factors (10, 11), this population is at higher risk of acquiring STIs or co-infections than are heterosexual men and women (CT/NG/TP: 20.0% in MSM vs. 4.2% in heterosexual men and women) (12, 13). A high proportion of sexually transmitted co-infections has been reported among MSM in North America (NG/CT: 20.4%) (14) and central Europe (CT/NG/TP: 20.0%) (12). In China, TP-related co-infections have been reported in various locations, such as Beijing (1.4 cases per 100-person years) (15), Chengdu (13.8%) (16), and Shenyang (22.9%) (17). Nonetheless, the limited number of STIs examined in these studies precluded the description of the detailed patterns of sexually transmitted co-infections among MSM. This limitation hampers the effective integration of prevention, screening, and treatment resources for different STIs, especially in low- and middle-income countries with a substantial STI burden and limited resources (18).

To close the abovementioned gap, we conducted a cross-sectional study in Shenyang, China, to assess the prevalence and describe the patterns of sexually transmitted co-infections among MSM, focusing on four STIs: HPV, CT, NG, and TP. A secondary aim of the study was to explore potential factors associated with increased STI acquisition.

## Methods

### Study Design and Data Collection

We conducted a cross-sectional study between July and December 2020 through a voluntary counseling and testing (VCT) clinic at The First Affiliated Hospital of China Medical University in Shenyang, China, to assess the prevalence and patterns of sexually transmitted co-infections among MSM. Participants who visited the VCT clinic were recruited if they were aged ≥18 years and agreed to participate in the study. After being informed of the study's purpose and protocol, they were inquired whether to give written consent or not. Obtained written consent, all participants were required to complete a self-administered digital questionnaire to collect potential factors associated with acquiring more STIs, including demographic and behavioral characteristics, such as age, ethnicity, educational background, the number of sexual partners, sexual behavior, etc. (English-version questionnaire is shown in Supplementary Material 1). The Checklist for Reporting Results of Cross-sectional Studies was utilized (see Supplementary Material 2).

### Collection and Detection of Specimens of HPV, CT, NG, and TP

We collected participants' both anal and genital specimens for HPV, CT, and NG testing and venous blood for TP testing. A skilled sampling technician was responsible for the collection of anal and genital specimens for HPV, CT, and NG testing. The elbow-knee position was applied to the process of anal sampling. After hands disinfection, the technician inserted a sterile swab into the anal canal with a depth of 5 cm, rotating left and right for around 20 s to successfully collect anal exfoliated cells. Genital samples were collected from the surface of genitals, coronary sulcus and urogenital tract secretions. The technician squeezed the urethra from the inside out and collected potential secretions. Collected anal and genital specimens were stored at −20°C and then transported to Kaipu Medical Laboratory in Shenyang, China, for fluorescence polymerase chain reaction analysis and detection for HPV, CT, and NG. To ensure the reliability of results from the third-party testing institutions, we set both negative and positive controls. Magnetic bead method was used for nucleic acid extraction. A total of 23 types of HPV were tested (types 6, 11, 16, 18, 31, 33, 35, 39, 42, 43, 44, 45, 51, 52, 53, 56, 58, 59, 66, 68, 73, 81, and 82). Venous blood TP testing was collected by a nurse and tested at The First Affiliated Hospital of China Medical University. We used rapid plasma reagin to screen TP, for which positive results were confirmed by *T. pallidum* particle assay. Individuals with positive results for both methods above were considered as current infection for TP. The same technician and nurse collected all anal and genital specimens and venous blood for consistency.

### Definitions

Participants in this study were classified according to STI status into four categories: (1) no infection; (2) single infection; (3) dual infection; (4) multiple infections. No infection was defined as having negative results for all four STIs (i.e., 23 genotypes of HPV, CT, NG, and TP).

### Sample Size Calculation

Based on the results of previous studies, we set the estimated sexually transmitted co-infections proportion among MSM at 30%, two-sided alpha at 0.05, and power at 0.8. We calculated the minimum sample size as 137 participants using the Power Analysis and Sample Size (PASS) software, version 15.0 (NCSS, LLC., Kaysville, UT, USA). Considering a 10% dropout rate, we expected a sample size of 151 participants.

### Statistical Analysis

For variables having missing values (<5%) from the questionnaire, we imputed the mean and mode for continuous and categorical variables, respectively. We used proportions for categorical variables and median and interquartile range for the age of participants. Given the ordinal trait of STI status, we considered the ordinal logistic regression analysis (proportional odds model) as the proper model to explore the associations between potential factors and the acquisition of more STIs. We performed the test of parallel lines to assess whether this model satisfies the proportional risk assumption. We also conducted collinearity diagnostics among all independent variables. Collinearity was considered if the model had a tolerance <0.1 or variance inflation factor >10. Finally, an ordinal logistic regression model was adopted. In the multivariable model, we included all variables with a *P* < 0.20 in the initial model to obtain adjusted odds ratios (aORs) and their 95% confidence intervals (CIs). Data were analyzed using SPSS Statistics, version 26.0 (IBM Corp., Armonk, NY, USA). A *P*-value <0.05 in the final model was considered statistically significant, and all reported *P*-values are two-sided.

## Results

### Characteristics of Participants

**Figure 1:**
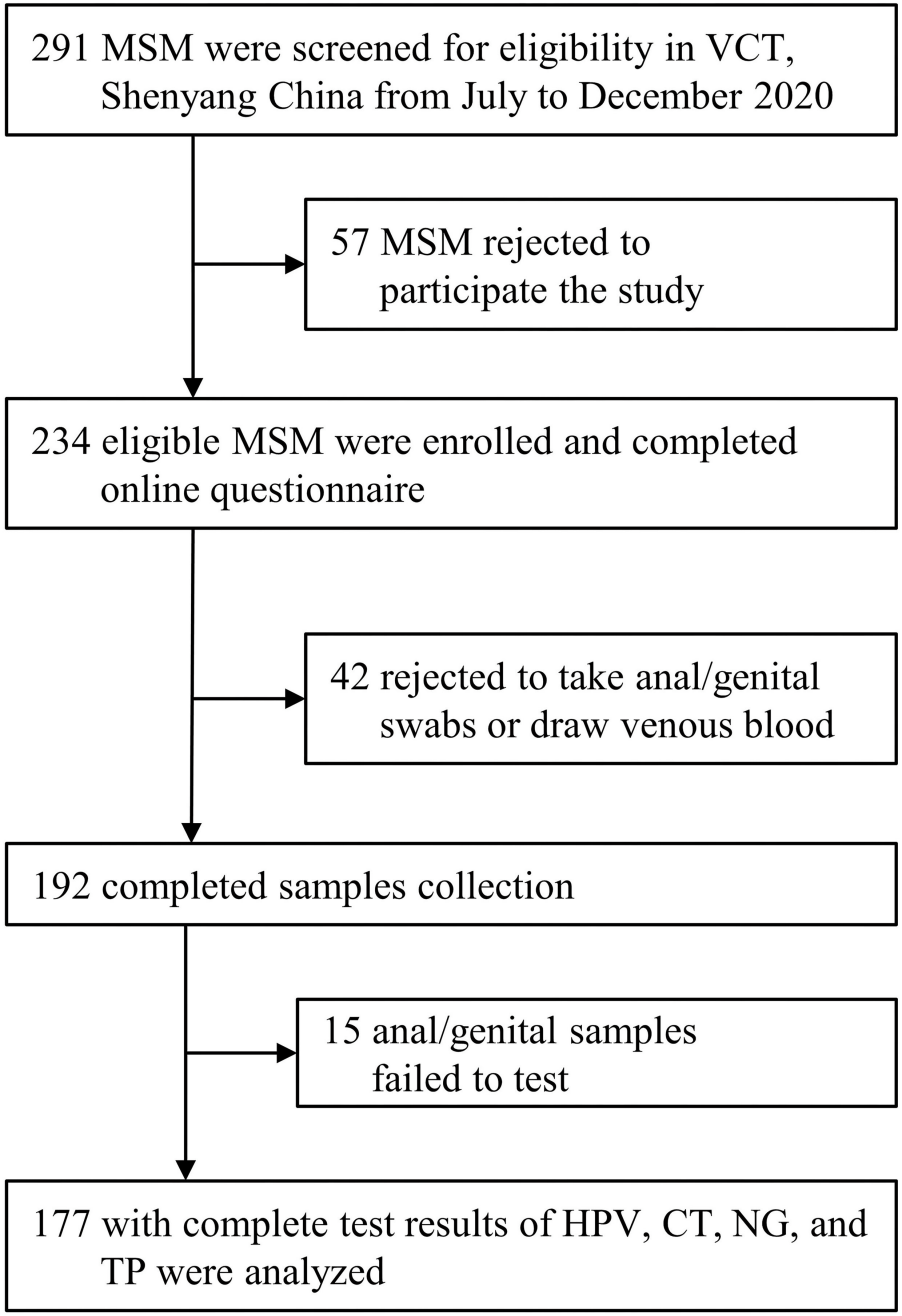
Inclusion flowchart. VCT, voluntary counseling and testing; HPV, human papillomavirus; CT, *Chlamydia trachomatis*; NG, *Neisseria gonorrhoeae*; TP, *Treponema pallidum*.

Of the 291 screened MSM who visited the VCT clinic, 234 were eligible and completed the digital questionnaire. Of the enrolled eligible MSM, the final analysis included 177 (75.6%) with complete results of tests for four STIs (HPV, CT, NG, and TP; Flowchart is presented in Figure 1). The median age of these participants was 29.0 (interquartile range: 23.0–38.0) years. Most participants had an educational background of undergraduate or above (108/177, 61.0%), were single (114/177, 64.4%), and had a monthly income ranging from US dollars $312–780 (89/177, 50.3%, Table 1). Additionally, 22.6% (40/177) of participants had a history of STIs, and 9.0% (16/177) were positive for HIV; however, only 55.4% (98/177) self-reported that they used condoms throughout the process of the last homosexual behavior.

**Table 1:** Demographic and behavioral characteristics among 177 MSM with no, single, dual, and multiple infection with STIs (HPV, CT, NG, TP).

	**Total**	**No infection**	**Single infection**	**Dual infections**	**Multiple infections**
	***N* (%)**	***n* (%)**	***n* (%)**	***n* (%)**	***n* (%)**
**Total**	177 (100)	30 (16.9)	105 (59.3)	34 (19.2)	8 (4.5)
**Age**					
18–29	90 (50.8)	17 (56.7)	48 (45.7)	21 (61.8)	4 (50.0)
30–39	52 (29.4)	9 (30.0)	34 (32.4)	5 (14.7)	4 (50.0)
40–49	19 (10.7)	2 (6.7)	13 (12.4)	4 (11.8)	0 (0.0)
50 or above	16 (9.0)	2 (6.7)	10 (9.5)	4 (11.8)	0 (0.0)
Median (IQR)	29.0 (23.0–38.0)	27.5 (22.8–37.3)	30.0 (25.0–38.0)	26.0 (20.8–39.5)	30.0 (19.3–38.5)
**Ethnicity**					
Han	149 (84.2)	25 (83.3)	90 (85.7)	28 (82.4)	6 (75.0)
Others	28 (15.8)	5 (16.7)	15 (14.3)	6 (17.6)	2 (25.0)
**Educational background**					
Senior high school or below	69 (39.0)	7 (23.3)	40 (38.1)	17 (50.0)	5 (62.5)
Undergraduate or above	108 (61.0)	23 (76.7)	65 (61.9)	17 (50.0)	3 (37.5)
**Working status**					
Full-time	85 (48.0)	13 (43.3)	58 (55.2)	10 (29.4)	4 (50.0)
Others	92 (52.0)	17 (56.7)	47 (44.8)	24 (70.6)	4 (50.0)
**Marital status**					
Single	114 (64.4)	23 (76.7)	64 (61.0)	23 (67.6)	4 (50.0)
Married	18 (10.2)	2 (6.7)	13 (12.4)	3 (8.8)	0 (0.0)
Others	45 (25.4)	5 (16.7)	28 (26.7)	8 (23.5)	4 (50.0)
**Monthly income (US dollar)**					
<312	45 (25.4)	10 (33.3)	23 (21.9)	11 (32.4)	1 (12.5)
312–780	89 (50.3)	10 (33.3)	54 (51.4)	18 (52.9)	7 (87.5)
More than 780	43 (24.3)	10 (33.3)	28 (26.7)	5 (14.7)	0 (0.0)
**The number of male sexual partners in the past 6 months**					
None	15 (8.5)	3 (10.0)	8 (7.6)	3 (8.8)	1 (12.5)
1–2	96 (54.2)	18 (60.0)	59 (56.2)	15 (44.1)	4 (50.0)
3 or above	66 (37.3)	9 (30.0)	38 (36.2)	16 (47.1)	3 (37.5)
**Condom use with male sexual partners in the past 6 months**					
Other	75 (42.4)	11 (36.7)	48 (45.7)	10 (29.4)	6 (75.0)
Every time	102 (57.6)	19 (63.3)	57 (54.3)	24 (70.6)	2 (25.0)
**Having sex with females in the past 6 months**					
No	148 (83.6)	27 (90.0)	84 (80.0)	32 (94.1)	5 (62.5)
Yes	29 (16.4)	3 (10.0)	21 (20.0)	2 (5.9)	3 (37.5)
**Drinking during or before sexual behaviors in the past 6 months**					
Never	131 (74.0)	24 (80.0)	76 (72.4)	25 (73.5)	6 (75.0)
Sometimes or more	46 (26.0)	6 (20.0)	29 (27.6)	9 (26.5)	2 (25.0)
**Substance use in the past 6 months**					
No	123 (69.5)	23 (76.7)	72 (68.6)	22 (64.7)	6 (75.0)
Yes	54 (30.5)	7 (23.3)	33 (31.4)	12 (35.3)	2 (25.0)
**Having tested for HIV**					
No	35 (19.8)	5 (16.7)	22 (21.0)	5 (14.7)	3 (37.5)
Yes	142 (80.2)	25 (83.3)	83 (79.0)	29 (85.3)	5 (62.5)
**The number of male insertive anal sex partners in the past 6 month**					
0	15 (8.5)	3 (10.0)	8 (7.6)	3 (8.8)	1 (12.5)
1–2	125 (70.6)	21 (70.0)	75 (71.4)	24 (70.6)	5 (62.5)
3 or above	37 (20.9)	6 (20.0)	22 (21.0)	7 (20.6)	2 (25.0)
**Having a history of STIs**					
No	137 (77.4)	27 (90.0)	82 (78.1)	24 (70.6)	4 (50.0)
Yes	40 (22.6)	3 (10.0)	23 (21.9)	10 (29.4)	4 (50.0)
**Condom use in the last homosexual behavior**					
Use throughout the process	98 (55.4)	22 (73.3)	56 (53.3)	19 (55.9)	1 (12.5)
Unused or discontinue use during the process	63 (35.6)	6 (20.0)	41 (39.0)	11 (32.4)	5 (62.5)
I forget it	16 (9.0)	2 (6.7)	8 (7.6)	4 (11.8)	2 (25.0)
**Sex role in the last homosexual behavior**					
Both	45 (25.4)	10 (33.3)	21 (20.0)	12 (35.3)	2 (25.0)
Insertive	64 (36.2)	11 (36.7)	40 (38.1)	9 (26.5)	4 (50.0)
Receptive	68 (38.4)	9 (30.0)	44 (41.9)	13 (38.2)	2 (25.0)
**Using PrEP for HIV**					
No	130 (73.4)	25 (83.3)	74 (70.5)	25 (73.5)	6 (75.0)
Yes	47 (26.6)	5 (16.7)	31 (29.5)	9 (26.5)	2 (25.0)
**HIV-positive**					
No	161 (91.0)	30 (100.0)	94 (89.5)	30 (88.2)	7 (87.5)
Yes	16 (9.0)	0 (0.0)	11 (10.5)	4 (11.8)	1 (12.5)

*IQR, interquartile range; MSM, men who have sex with men; PrEP, pre-exposure prophylaxis; STIs, sexually transmitted infections; HPV, human papillomavirus; CT, Chlamydia trachomatis; NG, Neisseria gonorrhoeae; TP, Treponema pallidum*.

### Prevalence and Patterns of STI Co-infections

**Figure 2:**
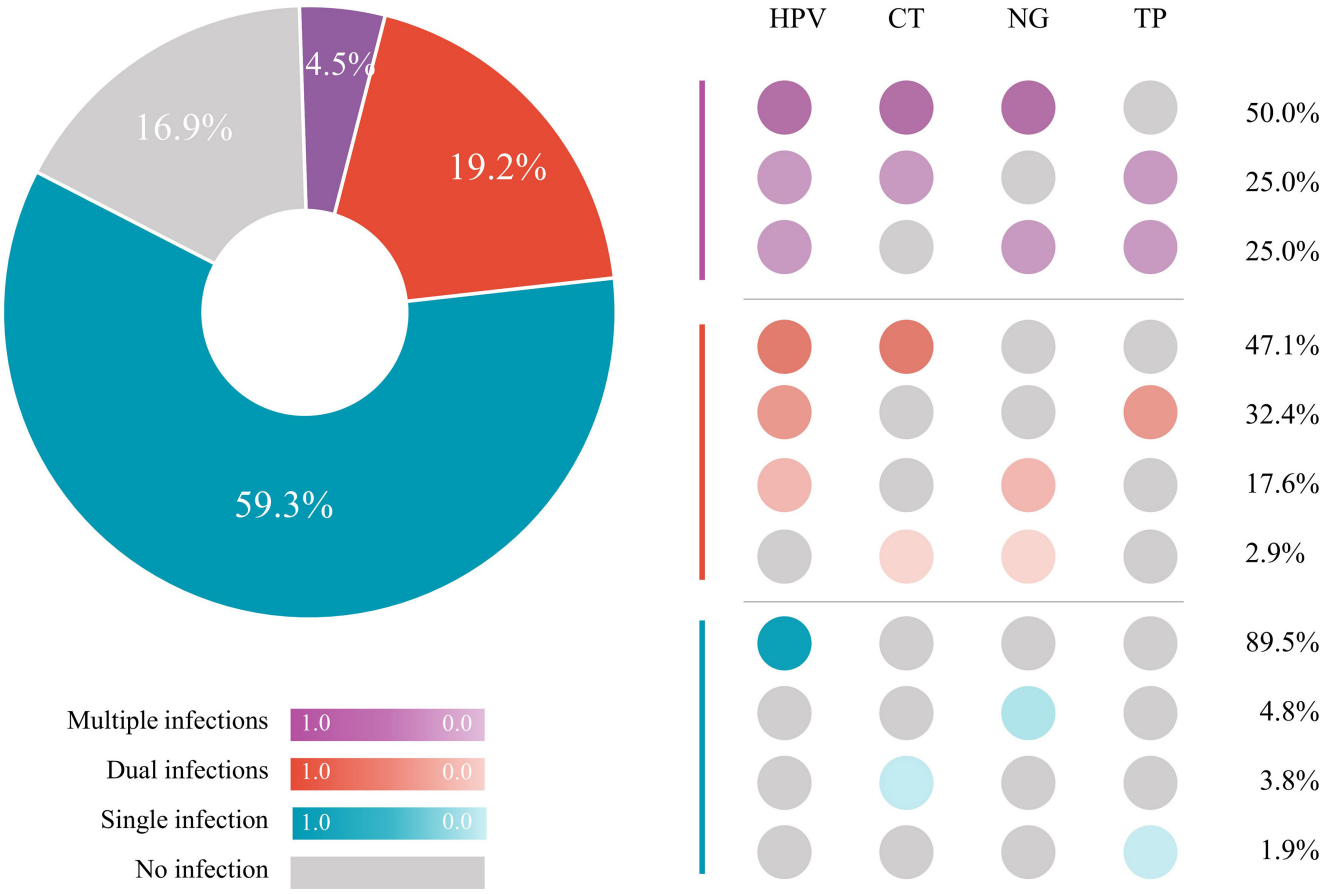
STI co-infections patterns among 177 MSM. HPV, human papillomavirus; CT, *Chlamydia trachomatis*; NG, *Neisseria gonorrhoeae*; TP, *Treponema pallidum*; STI, sexually transmitted infection; MSM, men who have sex with men.

Overall, 83.1% (147/177) of participants were infected with at least one of the four tested STIs (HPV, 76.3%; CT, 15.3%; NG, 10.2%; TP, 9.6%). The prevalence of HPV, CT, and NG in different sampling sites was presented in Table 2. Figure 2 shows the patterns of sexually transmitted co-infections and the prevalence of each pattern. Among the 177 participants, 105 (59.3%) had a single infection, 34 (19.2%) had dual infections, and eight (4.5%) had multiple (i.e., more than two) infections. The overall prevalence of co-infections was 23.7% (95% CI, 17.8%−30.8%). HPV/CT (47.1%) and HPV/CT/NG (50.0%) co-infection were the most prevalent types among participants with dual and multiple infections, respectively. Nearly all co-infections were related to HPV (97.1% of dual infections and 100% of multiple infections).

**Table 2:** The prevalence of HPV, CT, and NG stratified by sampling sites among 177 MSM.

**Category of STIs**	**Sampling sites**, ***n*** **(%)**	
	**Anal**	**Genital**
HPV	107 (60.5)	75 (42.4)
CT	17 (9.6)	13 (7.3)
NG	7 (4.0)	15 (8.5)

*STI, sexually transmitted infection; HPV, human papillomavirus; CT, Chlamydia trachomatis; NG, Neisseria gonorrhoeae*.

**Figure 3:**
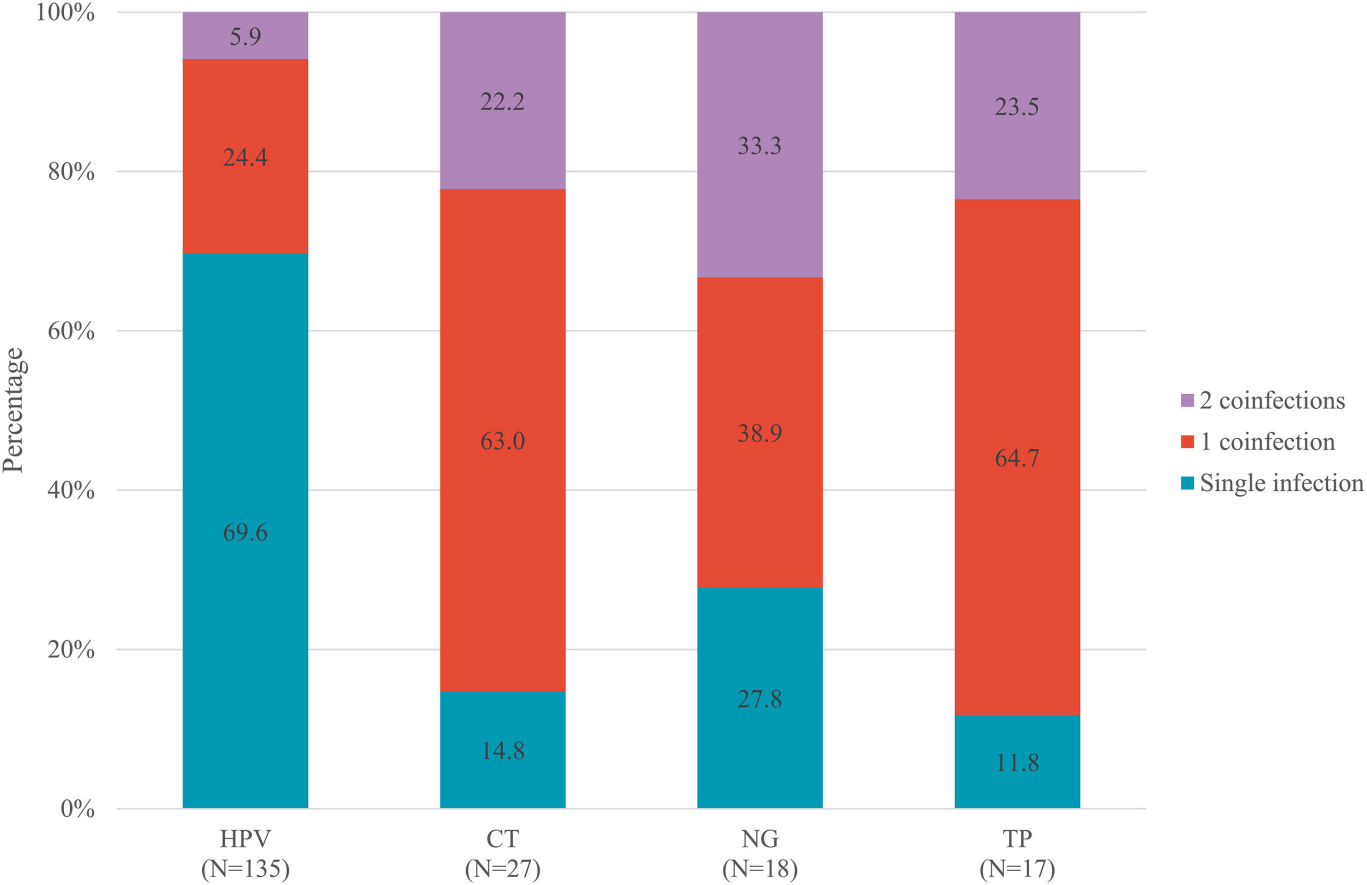
Prevalence of co-infections stratified by HPV, CT, NG, and TP, among 149 MSM with at least one STIs. HPV, human papillomavirus; CT, *Chlamydia trachomatis*; NG, *Neisseria gonorrhoeae*; TP, *Treponema pallidum*; STIs, sexually transmitted infections; MSM, men who have sex with men.

As presented in Figure 3, approximately 30.3% of participants with HPV, 85.2% of participants with CT, 72.2% of participants with NG, and 88.2% of participants with TP had a co-infection with at least one other STI. Despite the highest overall prevalence of HPV (76.3%), HPV-infected MSM had the lowest prevalence of co-infections (30.3%). Conversely, MSM infected with TP had the highest prevalence of co-infections (88.2%), of which 23.5% were co-infected with two other STIs.

**Figure 4:**
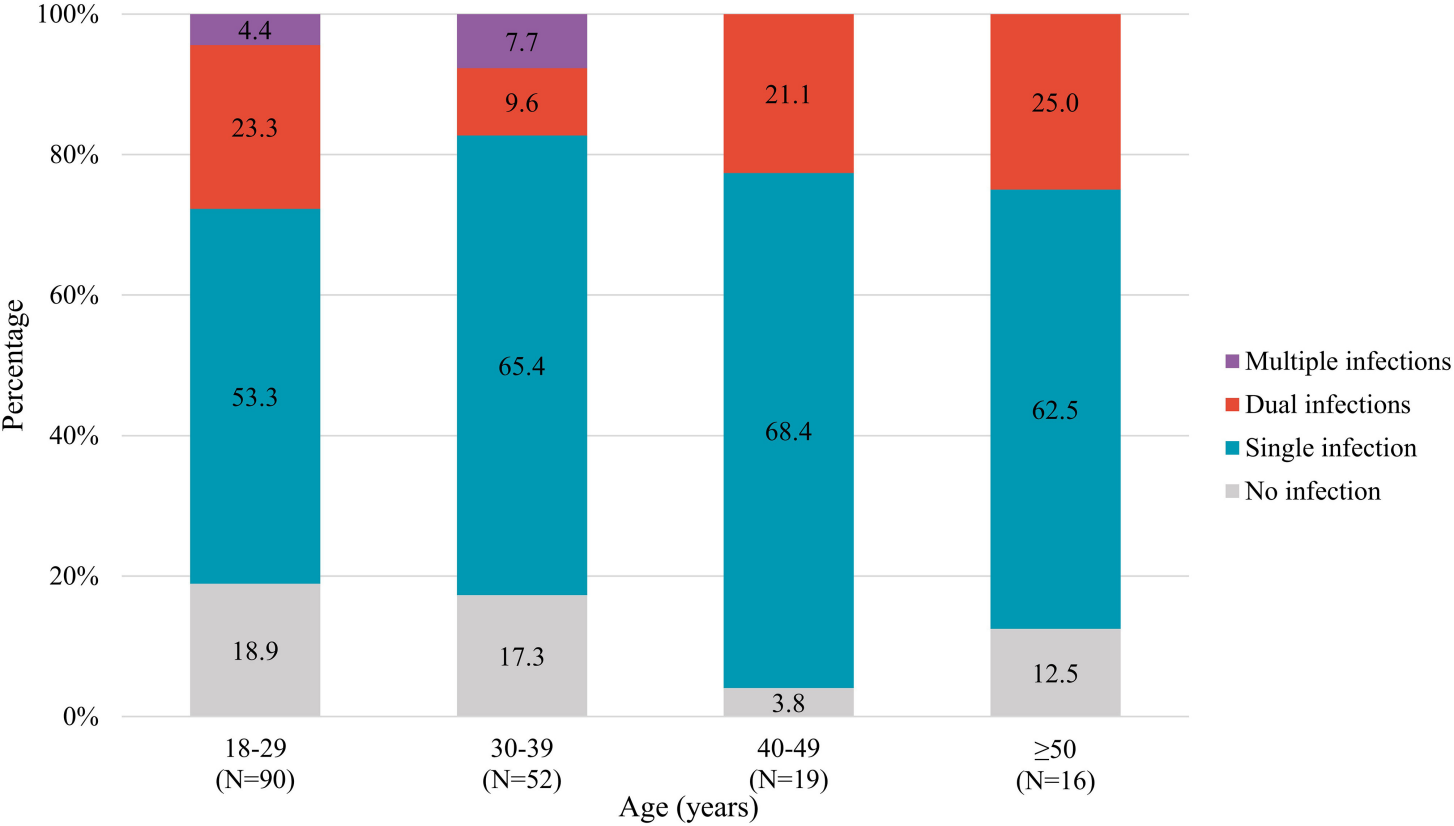
STI co-infections patterns stratified by age groups among 177 MSM. STI, sexually transmitted infection; MSM, men who have sex with men.

In addition, patterns of STI co-infections in diverse age subgroups are presented in Figure 4. The prevalence of STI co-infections among MSM aged 18–29, 30–39, 40–49, and ≥50 years was 27.7, 17.3, 21.1, and 25.0%, respectively. No significant difference was observed among the diverse age groups. However, we found individuals co-infected with more than two STIs only among younger MSM.

### Factors Associated With More STIs Acquisition

In univariable analysis, several variables were significantly associated with acquiring more STIs: educational background, history of STIs, using condoms in the last homosexual behavior, and living with HIV. In multivariable ordinal logistic regression analysis, factors that remained significant in the final model were higher educational level (aOR, 0.46; 95% CI, 0.24–0.85; *P* = 0.014) and the history of STIs (aOR, 2.53; 95% CI, 1.24–5.18; *P* = 0.011; Table 3).

**Table 3:** Ordinal logistic regression analysis for factors associated with acquiring more STIs among 177 MSM.

**Risk factors**	**Univariable analysis**			**Multivariable analysis**		
	**cOR**	**95% CI**	***P*-Value**	**aOR**	**95% CI**	***P*-Value**
**Educational background**						
Senior high school or below	Ref.	Ref.		Ref.	Ref.	
Undergraduate or above	0.46	0.25–0.84	0.011	0.46	0.24–0.85	0.014^*^
**Having a history of STIs**						
No	Ref.	Ref.		Ref.	Ref.	
Yes	2.38	1.19–4.78	0.014	2.53	1.24–5.18	0.011^*^
**Condom use in the last homosexual behavior**						
Use throughout the process	Ref.	Ref.		Ref.	Ref.	
Unused or discontinue use during the process	1.82	0.97–3.41	0.061	1.81	0.96–3.43	0.069
I forget it	2.62	0.91–7.54	0.075	2.86	0.98–8.35	0.055
**HIV-positive**						
No	Ref.	Ref.		Ref.	Ref.	
Yes	2.10	0.81–5.42	0.127	1.34	0.49–3.63	0.570

*cOR, crude odds ratio; aOR, adjusted odds ratio; CI, confidence interval; MSM, men who have sex with men; STIs, sexually transmitted infections. All variables with a P < 0.20 were included in adjusted regression model*.

* *P < 0.05*.

## Discussion

### Principal Findings and Significance

Given limited epidemiological data on patterns of STI co-infections among MSM, we estimated the prevalence and elucidated co-infection patterns of four STIs (HPV, CT, NG, and TP) among MSM. Potential factors associated with acquiring more STIs were also explored. We found that more than 80% of MSM enrolled in this study had an STI burden, of whom nearly 30% had a sexually transmitted co-infection. HPV/CT and HPV/CT/NG co-infections predominated among participants with dual and multiple infections, respectively. We also found that lower educational level and a history of STIs were potential risk factors for acquiring more STIs. These epidemiological data shed light on the micro and macro evidence that could help public health policymakers optimize current STI prevention, screening, and treatment strategies, particularly in low- and middle-income countries with limited health resources (18).

### Prevalence and Patterns of STI Co-infections

Our findings in this study showed that MSM suffer a considerable burden of overall STIs and co-infections. As more STIs were analyzed in this study, the overall STI and co-infection prevalence among MSM was higher than previously reported data from 13.8 to 22.9%. This indicates the limitations of previous research in revealing the heavy burden of sexually transmitted co-infections among MSM. More attention should thus be paid to sexually transmitted co-infections among MSM.

The pattern we reported for four STIs (HPV/CT/NG/TP) was similar to that of a study in South Africa (19): the prevalence of HPV was the highest, followed by CT, NG, and TP. The predominant co-infection type was also related to HPV, which could be attributed to the fact that HPV infections can persist for life and be detected easier than other bacterial STIs. Persistent infection with these two organisms may also elevate the risk of acquiring other STIs as a result of exacerbated inflammation (5, 7). Vaccination against HPV, therefore, should be the priority rather than treatment of symptoms. Furthermore, ~90% of participants infected with TP had a co-infection with another STI, of which more than 20% were co-infected with ≥2 other STIs. This suggests that screening and prevention of other STIs are necessary in an individual following a positive TP test and could yield positive outcomes. Previous research revealed that single STI testing is accompanied by a high proportion of missed diagnoses for other STIs (20) and that MSM continue to be underdiagnosed (21). Given these observations and the woefully inadequate health resources per capita in China, an optimal STI co-testing strategy integrated with prevention and treatment services for MSM is warranted and feasible.

### Factors Associated With More STIs Acquisition

In addition to assessing the prevalence of co-infections, it is also critical to identify characteristics associated with acquiring more STIs. We found that among MSM, lower educational level and the history of STIs were potential risk factors for acquiring more STIs. In this study, less-educated MSM (22/69, 31.9%) bore a heavier burden of sexually transmitted co-infections than well-educated MSM (20/108, 18.5%); notwithstanding, relatively less attention was paid to this population in most previous work. It has been reported that lower educational level is associated with STIs acquisition among general population (22, 23), which is similar to our results. Compared with well-educated individuals, those with lower educational levels may lack awareness of self-protection from STIs; they may engage with riskier partners and sexual behaviors, increasing risk of STIs infection. Furthermore, MSM with lower educational levels had fewer testing behaviors (24) and always have a test for STIs symptom, whereas among higher educated individuals, testing behaviors are performed for being worried about their health (23). Focus should also center on MSM with a history of STIs (25). These individuals may be more vulnerable to other STIs because of the role of chronic and refractory inflammation; even if an acute bacterial infection is treated, the genital tract milieu might be perturbed for perhaps months or longer, resulting in residual inflammation (26). In the final model, unprotected homosexual intercourse showed a marginal statistical significance on STIs acquisition. Low education was reported to be associated with unprotected anal intercourse with new partner (27), which may expand the transmission of STIs. Although we failed to found the impact of age on STIs infection, individuals co-infected with more than two STIs only existed in younger MSM. Younger individuals may bear higher incidence of STIs but have inadequate healthcare engagement (28, 29), which will impede the prevention for STIs. Appropriate attention should be paid to these vulnerable population, such as targeted education regarding prevention measures, especially persistent condom use.

Finally, it is also critical to obtain accurate STI surveillance data to develop a prevention and control program and institute measures to adequately evaluate the effect of behavioral interventions and measure the likelihood of STI transmission. However, STI surveillance systems are often limited in the developing world (30); only HIV, NG, and TP are reportable STIs in the national surveillance system in China (31). Effective utilization of information regarding co-infections involving currently reportable STIs will promote the targeted distribution of limited health resources.

### Limitations

Due to the limited number of samples and single recruitment method, selection bias is inevitable in this study, thus limiting the extrapolation of results to the general MSM population. Our results regarding sexually transmitted co-infections from the cross-sectional analysis failed to reveal the time sequence of acquisition of each STI. Prospective studies are needed to clarify the sequential or concurrent relationship regarding acquiring these infections. The self-reported data on demographic and behavioral characteristics are susceptible to both recall and social desirability biases. Due to the cross-sectional design, this study is merely able to explore associations rather than determine causality. However, a significant strength of this study is the complete data on four STIs, which allowed the estimation of detailed patterns of co-infections among MSM. This study thus represents a vital reference for health policymakers to address suboptimal prevention, screening, and treatment strategies.

## Conclusions

MSM in Shenyang—particularly less-educated individuals—suffer a substantial burden of sexually transmitted co-infections. Preventive services should focus more on MSM with lower educational levels and a history of STIs. An optimized multi-STI co-testing strategy integrated with prevention, surveillance, and treatment is urgently needed to reduce the prevalence of sexually transmitted co-infections in resource-limited countries.

## Data Availability Statement

The raw data supporting the conclusions of this article will be made available by the authors, without undue reservation.

## Ethics Statement

The studies involving human participants were reviewed and approved by the Institutional Review Board Committee of the First Affiliated Hospital of China Medical University in Shenyang, China [(2020) 2015-140-6]. The patients/participants provided their written informed consent to participate in this study.

## Author Contributions

Q-HH conceived and designed the study. Z-HY, FL, S-TC, and Z-ZL collected the data for the study. Z-HY and SC analyzed the data and interpreted the results. Z-HY wrote the first draft of the manuscript. Y-JJ and Q-HH revised the manuscript. All authors have reviewed and approved the final manuscript.

## Funding

This work was funded by the National Natural Science Foundation of China (82073620).

## Conflict of Interest

The authors declare that the research was conducted in the absence of any commercial or financial relationships that could be construed as a potential conflict of interest.

## Publisher's Note

All claims expressed in this article are solely those of the authors and do not necessarily represent those of their affiliated organizations, or those of the publisher, the editors and the reviewers. Any product that may be evaluated in this article, or claim that may be made by its manufacturer, is not guaranteed or endorsed by the publisher.
